# Dental Engagement Book

**Published:** 1860-02

**Authors:** 


### DENTIST ENGAGEMENT BOOK.

We have received an engagement book, which was gotten up and published by Jones & White, of Philadelphia.

It is better arranged than any thing we have before seen, for this purpose, the days of the week and hours of the day only are given, and not the month or day of the month, or the year, either or all of these can be added if necessary.

By this arrangement every page can be occupied.

The day and hour is given, so that it is only necessary to place the name of the patient opposite the hour of the day for which the engagement is made.

We have tested it, and our advice is, *try it.*

For sale at the Dental Depots. Price 75 cents. T.

